# Subclavian vein aneurysm: A case report of a very rare vascular anomaly with a comprehensive literature review

**DOI:** 10.1097/MD.0000000000043278

**Published:** 2025-07-18

**Authors:** Hamza A. Abdul-Hafez, Alaa Zayed, Abdullah Raed Hawawrah, Mohammed A. Barakat, Alaa Hasan, Mahmoud Alawneh

**Affiliations:** a Department of Medicine, Faculty of Medicine and Health Sciences, An-Najah National University, Nablus, Palestine; b Department of Radiology, Rafidia Surgical Hospital, Nablus, Palestine.

**Keywords:** aneurysm, case reports, subclavian vein, vascular, vascular anomaly

## Abstract

**Rationale:**

Subclavian vein aneurysms are an exceedingly rare vascular anomaly, often diagnosed incidentally due to their nonspecific or absent clinical manifestations. Due to the limited number of reported cases and the lack of standardized diagnostic and management protocols, clinical decision-making remains challenging. This report presents a rare incidental case managed conservatively and is accompanied by a comprehensive literature review to consolidate existing knowledge on clinical presentation, associated risk factors, imaging modalities, and management approaches. By synthesizing available data, this study aims to support clinicians in recognizing this unusual entity and guide evidence-informed, individualized management strategies in the absence of formal guidelines.

**Patient concern:**

A 37-year-old previously healthy male presented with persistent posterior neck pain for 1 month.

**Diagnosis:**

Imaging studies, including magnetic resonance angiography and computed tomography angiography, identified a fusiform aneurysmal dilation of the right subclavian vein measuring 2.2 × 2.0 × 1.7 cm.

**Intervention:**

A multidisciplinary team decided on conservative management, focusing on lifestyle modifications, analgesics, and closed follow-up, as the aneurysm was stable and asymptomatic regarding vascular complications.

**Outcome:**

During follow-up at 3, 6, and 12 months, the patient experienced gradual symptom improvement. Imaging confirmed the aneurysm remained stable without complications.

**Lessons:**

This case highlights the importance of considering subclavian vein aneurysms in the differential diagnosis of neck pain and supraclavicular masses. It further emphasizes the important role of imaging in their identification and monitoring. By reviewing previously reported cases, this study contributes valuable insights into their clinical presentation, diagnostic modalities, and management strategies.

## 1. Introduction

Venous aneurysms are rare clinical entities that have been reported in nearly all parts of the venous system, including the extremities, the superior and inferior vena cava, and the head and neck regions.^[1]^ The exact etiology of venous aneurysms remains unclear, but they are generally classified into 3 main categories: congenital, acquired, and traumatic.^[2]^

Subclavian vein aneurysms (SVAs) are focal dilations of the subclavian vein, most commonly of congenital origin. Patients with SVA can present with a variety of clinical presentations, ranging from neck pain and swelling, which enlarge with the Valsalva maneuver, to asymptomatic, typically discovered incidentally during imaging studies. While SVAs are generally benign, they can lead to serious complications such as thromboembolism, rupture, venous obstruction, and compression of surrounding structures, such as neurovascular structures, resulting in neurological symptoms.^[2,3]^

SVAs are exceedingly rare, with only a few documented cases in the literature. Our review identified only 13 previously reported cases.^[2–14]^ Despite many cases and reviews of other peripheral venous aneurysms, SVAs have been poorly studied. So, we aim to provide a comprehensive review of previously reported cases in the literature, including demographic characteristics of the patients, risk factors and associated conditions, clinical presentation, diagnostic modalities, and management and outcomes.

Here, we describe the case of a 37-year-old previously healthy patient who presented with posterior neck pain. During the diagnostic workup, an SVA was incidentally discovered. The patient was successfully managed with conservative therapy and demonstrated a favorable long-term outcome without complications.

## 2. Case presentation

A 37-year-old previously healthy male presented to the outpatient clinic with a complaint of posterior neck pain lasting for 1 month. He had been prescribed muscle relaxants and paracetamol after a cervical X-ray showed signs of muscle spasm. However, 2 weeks later, his pain persisted despite the treatment, prompting him to seek further medical attention at the neurology clinic. The patient denied any history of trauma, invasive procedures, or surgery to the neck. He also did not report neck swelling, supraclavicular pain, or symptoms such as upper limb weakness or numbness. His medical and surgical history was unremarkable, and there was no family history of vascular or congenital disorders.

On examination, the patient’s vital signs were stable, with a blood pressure of 125/80 mm Hg, heart rate of 86 bpm, and oxygen saturation of 98%. He was afebrile. Inspection of the neck revealed no swelling, bruising, or tenderness. Neurological examination showed normal symmetrical muscle strength (5/5) in both upper and lower limbs, with intact sensation and reflexes. Peripheral pulses were also intact. On systemic examination, the cardiovascular assessment revealed a regular rate and rhythm, normal S1 and S2 without murmurs or gallops, and no peripheral edema. The respiratory examination showed clear breath sounds bilaterally with no crackles, wheezes, or pleural rub. Abdominal examination was unremarkable; the abdomen was soft, nontender, with normal bowel sounds and no hepatosplenomegaly. Skin examination showed no rashes, ecchymoses, cyanosis, or digital clubbing. Baseline laboratory tests, including complete blood count, coagulation profile, and liver and renal function, were all within normal limits.

**Figure 1.**
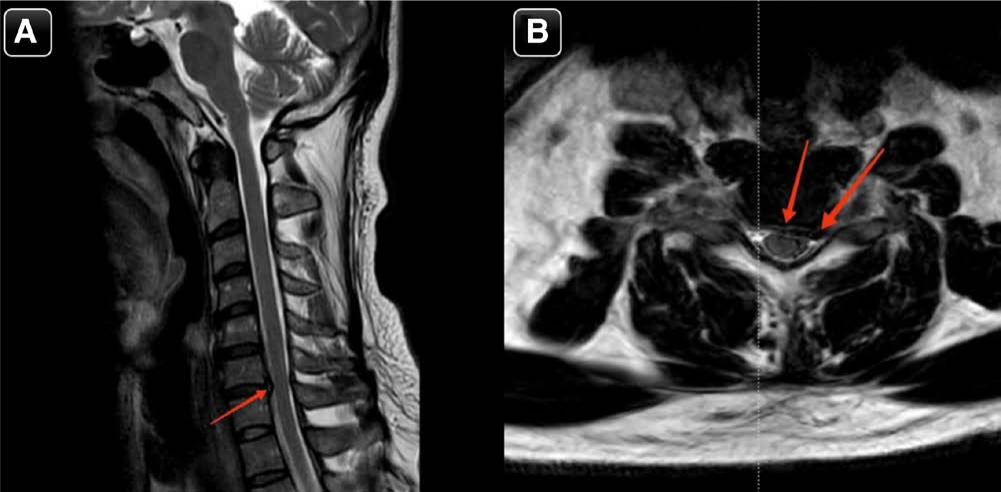
(A & B) Coronal reconstruction of MRA/MRV of the neck showing aneurysmal dilatation of the right subclavian vein (yellow arrow). MRA = magnetic resonance angiography;

Cervical magnetic resonance imaging (MRI) demonstrated loss of the normal cervical lordosis, likely due to muscle spasm. It also revealed a right paracentral disc protrusion at the C5/C6 level, indenting the anterior subarachnoid space. Mild disc bulging with a left paracentral/posterolateral disc protrusion and osteophyte complex at the C6/C7 level caused mild mass effect on the ventral aspect of the spinal cord. Additionally, there was mild secondary narrowing of the central canal at C6/C7, but no significant stenosis (Fig. 1). No other focal cervical disc herniations were observed.

**Figure 2.**
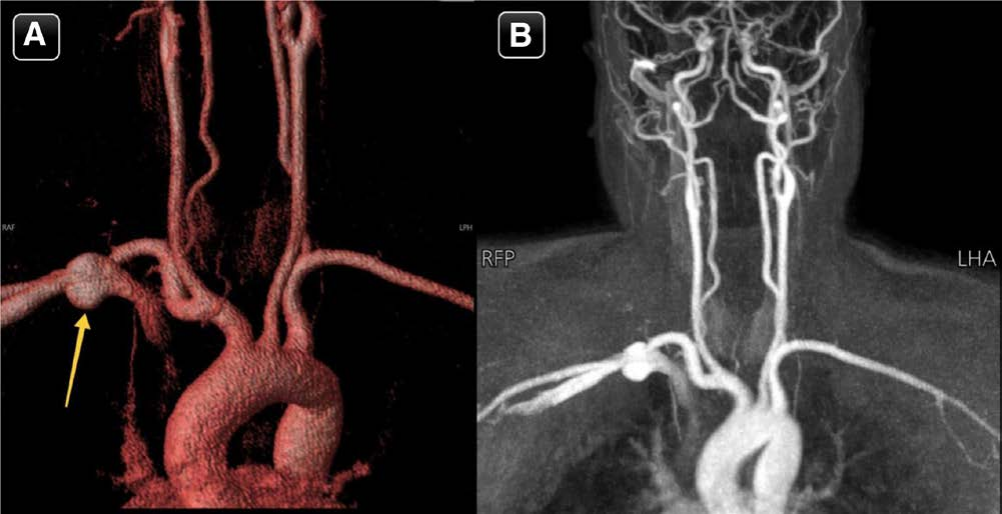
(A & B) Sagittal and axial sections of T2WI cervical spine MRI showing mild diffuse disc bulge more to the left side (red arrows) causing mild central canal and left neural foraminal stenosis. magnetic resonance imaging

Magnetic resonance angiography of the neck and brain revealed an abnormal rounded signal at the proximal right subclavian vein, suggestive of venous aneurysmal dilation (Fig. 2). The course, caliber, and branching of the internal carotid arteries, vertebrobasilar system, and the arterial components of the Circle of Willis were normal, with no evidence of vascular anomalies, aneurysms, stenotic segments, occlusions, or deviations. The common carotid arteries, both extracranial internal and external carotid arteries, and extracranial vertebral arteries also demonstrated normal caliber and branching.

**Figure 3.**
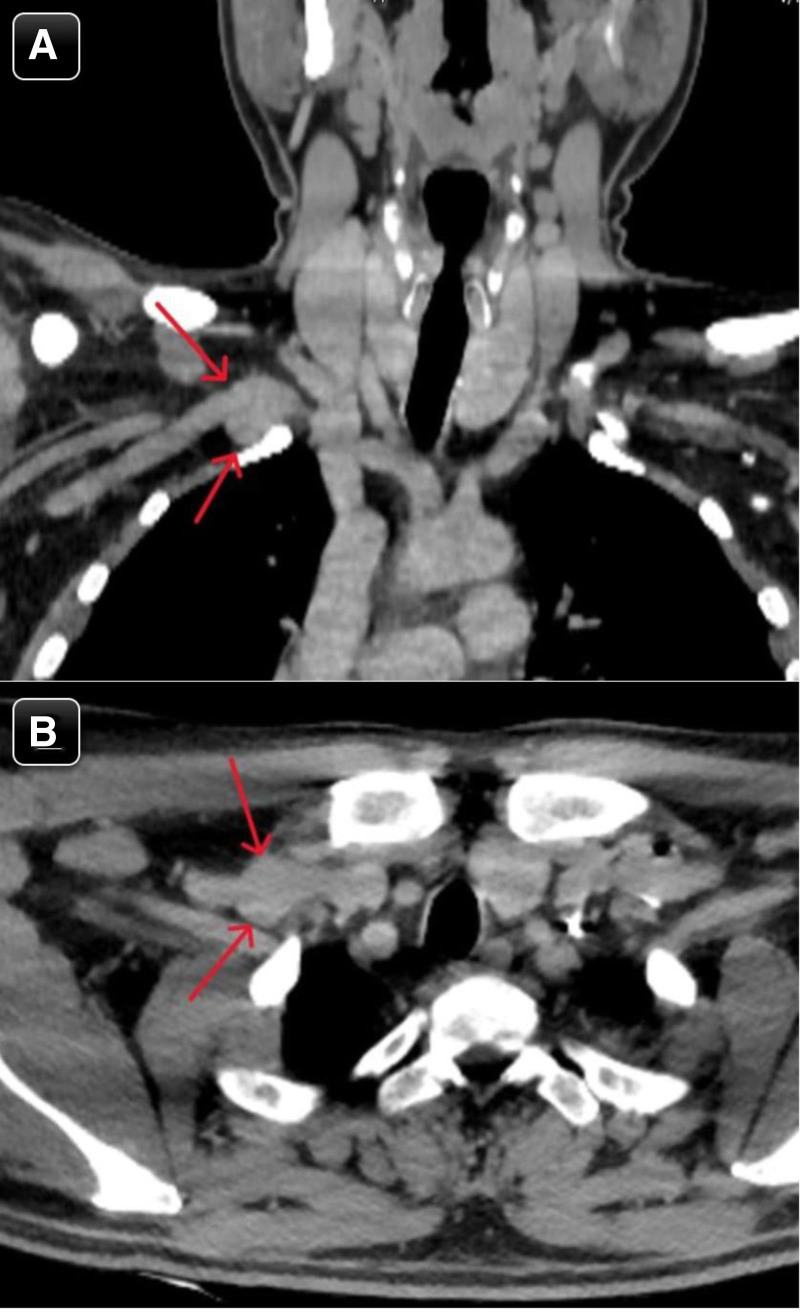
(A & B) Coronal and axial view of neck CT angiography showed aneurysmal fusiform dilation of the proximal right subclavian vein.

For further evaluation of the vascular findings, a neck computed tomography (CT) angiography was performed, which confirmed a fusiform aneurysmal dilation of the proximal right subclavian vein, measuring 2.2 × 2.0 × 1.7 cm (Fig. 3). The CT angiography also showed the normal caliber, outline, and anatomical configuration of the aorta and other vessels, with no evidence of dissection or aneurysm.

After a multidisciplinary discussion, conservative management was pursued for the patient’s cervical pain. He was prescribed oral paracetamol 1 g every 8 hours and ibuprofen 400 mg as needed for pain control. He was also given work-related advice, including improving posture and avoiding neck strain. His symptoms improved gradually with this approach. No anticoagulation was given, as the aneurysm was stable and without signs of thrombosis. It should be emphasized that the patient’s posterior neck pain was secondary to muscle spasm and cervical disc pathology; the SVA was an incidental finding and did not contribute to his presenting symptoms.

At follow-up visits at 3, 6, and 12 months, the patient remained clinically stable and reported a gradual improvement in his neck pain. He denied any complications related to the vascular aneurysm. A follow-up CT angiography report revealed that the size of the right-sided SVA remained stable.

## 3. Results

Table 1 shows a descriptive analysis of 13 patients with SVA identified in the literature. The mean age was 49.92 ± 25.44 years, with a marked female predominance (10/13, 76.9%). Aneurysm size varied widely, ranging from 1.7 × 0.8 cm to 15 × 10 cm (mean: 4.51 ± 3.13 cm). There was a slight left-sided predominance (7/13, 53.8%) compared with right-sided cases (5/13, 46.2%) (Table 1).

**Table 1:** Descriptive review of previous reported cases of subclavian vein aneurysm.

Characteristics	N (%)	Characteristics	N (%)
Total number of patients	13	Clinical presentation	
Age (years)		Asymptomatic	5 (38.4)
Mean (± SD)	49.92 (25.44)	Neck pain	4 (30.7)
Gender		Neck swelling	8 (61.5)
Male	3 (23.1)	SOB	1 (7.6)
Female	10 (76.9)	Dysphagia	1 (7.6)
Size (cm)		Hoarseness	1 (7.6)
Mean (± SD)	4.51 (3.13)	Stroke-like symptoms	1 (7.6)
Largest	15 × 10	Investigation	
Smallest	1.7 × 0.8	CT	13 (100)
Side		MRI	1 (7.6)
Right	5 (46.2)	Ultrasound	6 (46.1)
Left	7 (53.8)	Histopathology	7 (53.8)
Risk factors and associated diseases		Management	
HTN	3 (23.1)	Conservative	5 (38.4)
DM	2 (15.3)	Open aneurysmectomy	6 (46.1)
Previous interventional procedures	2 (15.3)	Endovascular	3 (23.1)
Neck trauma	2 (15.3)	Complications	
Other vascular anomalies	4 (30.7)	Thrombosis	2 (15.3)
NF-1	1 (7.6)		
Follow-up			
Favorable outcomes	11 (84.6)		

CT = computed tomography, DM = diabetes mellitus, HTN = hypertension, MRI = magnetic resonance imaging, NF-1 = neurofibromatosis type 1, SOB = shortness of breath.

**Figure 4.**
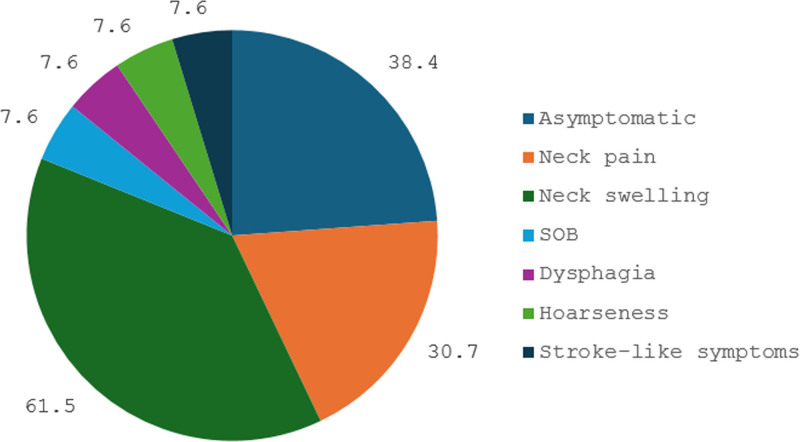
Pie chart illustrating the clinical presentation of subclavian vein aneurysms in 13 reported cases. SOB = shortness of breath.

Clinical presentation of SVAs varies widely, including 5 patients (38.4%) who were asymptomatic, whereas the majority exhibited neck swelling (8/13, 61.5%) or neck pain (4/13, 30.7%). Less frequent presentations included shortness of breath, dysphagia, hoarseness, and stroke-like symptoms, each reported in 1 patient (7.6%) (Table 1 and Figure 4).

Radiological imaging plays an important role in the diagnosis of SVAs. CT scan was the most frequently used modality, which was used in all cases (13/13, 100%), followed by ultrasound (US) in 6 cases (46.1%) and MRI in 1 case (7.6%), while histopathological examination was performed in 7 patients (53.8%). Documented risk factors and associated conditions comprised hypertension in 3 cases (23.1%), diabetes mellitus in 2 (15.3%), prior interventional procedures in 2 (15.3%), history of neck trauma in 2 (15.3%), other vascular anomalies in 4 (30.7%), and neurofibromatosis type 1 in 1 (7.6%) (Table 1).

Management strategies varied according to symptoms and perceived risk. Conservative management was selected for 5 patients (38.4%), typically those asymptomatic or considered low risk, with reported stability on follow-up. Open aneurysmectomy was performed in 6 patients (46.1%), often in symptomatic or larger aneurysms, with no recurrence reported in the available follow-up. Endovascular interventions were used in 3 cases (23.1%). Thrombosis was reported as a complication in 2 patients (15.3%). Follow-up findings indicated favorable outcomes in 11 patients (84.6%), reflecting stability or resolution without major adverse events over the reported follow-up periods (Table 1).

## 4. Discussion

The differential diagnosis for posterior neck pain is broad and includes musculoskeletal, neurologic, infectious, neoplastic, and vascular causes. In this case, the initial evaluation focused on common musculoskeletal etiologies, such as cervical muscle strain, facet joint arthropathy, and degenerative disc disease, which are among the most frequent causes of neck pain.^[15,16]^ MRI findings confirmed cervical disc protrusions at the C5–C6 and C6–C7 levels, along with loss of normal lordosis, consistent with muscle spasm and discogenic pain. Cervical radiculopathy and myelopathy were considered but ruled out clinically and radiologically due to the absence of neurological deficits.^[17]^ Other potential causes, including neoplastic involvement, inflammatory conditions, or referred pain from visceral or vascular sources, were considered less likely given the clinical stability, lack of systemic symptoms, and normal systemic examination.^[17,18]^

During the vascular imaging workup performed to exclude possible compressive or structural vascular causes, a fusiform dilation of the right subclavian vein was incidentally identified. While not related to the patient’s symptoms, it raised concern for entities such as a true venous aneurysm, either idiopathic or secondary to trauma, congenital wall weakness, or prior instrumentation, as well as pseudoaneurysm, venous ectasia, low-flow vascular malformation, or post-thrombotic change.^[2]^ Arterial causes, including subclavian artery aneurysm or Kommerell diverticulum, were also considered and excluded with imaging.

Venous aneurysms are rare vascular anomalies characterized by a localized dilation of a vein involving all 3 layers of the vessel wall.^[19]^ Unlike arterial aneurysms, which are more common, venous aneurysms can develop throughout the venous system and are classified as either deep or superficial, depending on the affected vein.^[19,20]^ Although uncommon, venous aneurysms were first described by Osler et al^[21]^ in 1913. Since then, numerous case reports have documented their occurrence in various locations throughout the body, despite the limited literature on the subject.

Among these, thoracic venous aneurysms represent a particularly rare subset, involving abnormal dilations within the venous structures of the thoracic cavity. They can affect several major veins, including the brachiocephalic, innominate, azygos, and superior vena cava.^[19,22]^ Aneurysms of the subclavian vein, which are part of this group, remain exceedingly rare and can present with a variety of clinical signs. We provide a comprehensive review and description of previous reported cases of SVA to provide further insight into their clinical presentation, risk factors, diagnostic modalities, and management of this rare vascular anomaly (Table 1 and Table S1, Supplemental Digital Content, http://links.lww.com/MD/P514).

Clinical presentation of SVA varies widely, with some patients remaining asymptomatic while others experience significant symptoms. In previously reported cases, the majority of cases presented with neck swelling, followed by neck pain. Additional symptoms included shortness of breath, dysphagia, hoarseness, and stroke-like neurological complaints, suggesting that larger aneurysms or those in restrictive anatomical locations may cause compressive effects on adjacent structures. Physical examination often revealed a soft, compressible supraclavicular mass, which became more prominent with the Valsalva maneuver, a key diagnostic feature suggesting venous origin.

Radiological imaging, including CT scan, US, and MRI, has a crucial role in the diagnosis of SVAs. These imaging techniques not only confirm the presence of an aneurysm but also help assess its size, extent, and potential complications.^[2]^ Histopathological examination was performed in several cases, revealing changes such as venous wall thickening, thrombus formation, and hamartomatous alterations, further supporting the diagnosis when tissue analysis was available.

The underlying etiology of SVAs remains unclear, though certain factors may contribute to their development. Hypertension was the most frequently reported comorbidity, followed by diabetes and prior interventional procedures, suggesting a potential role for iatrogenic trauma in aneurysm formation. Additionally, congenital or genetic factors may play a role, such as neurofibromatosis type 1. A history of trauma was identified in 2 cases, supporting the hypothesis that endothelial injury may be a contributing factor. Other vascular anomalies, including venous malformations and hamartomas, were present in 4 cases, suggesting a possible congenital component in select cases.

Given their rarity, SVAs do not have well-established management guidelines, and treatment is typically individualized based on symptomatology and risk factors. Among reported cases, open surgical intervention was the most frequently chosen management strategy, performed in 6/13 (46.1%), particularly in symptomatic cases or when thromboembolic risk was a concern. Surgical excision remains the preferred approach, especially when the aneurysm is large or symptomatic.^[2]^ Conservative management was pursued in 5/13 (38.4%), typically in asymptomatic patients or those deemed to have a lower risk of complications.

Endovascular surgical treatment was performed in 3 cases. One patient who underwent endovascular treatment continued to experience symptoms three weeks later, necessitating an aneurysmorrhaphy for stent excision and aneurysm resection.^[7]^ Another patient who underwent transcatheter coil embolization later developed thrombosis and pulmonary embolism as a post-procedure complication, raising concerns about the safety of minimally invasive approaches in certain cases. While endovascular techniques such as stent placement and coil embolization are considered alternatives for patients who are not candidates for open surgery, their use remains limited, and complications can pose significant risks.^[11]^

Follow-up duration, when available, ranged from 2 to 18 months, with no recurrence noted in most cases. These findings suggest that conservative management may be appropriate for select cases, while surgical or endovascular interventions should be considered for symptomatic or high-risk aneurysms. However, given the rarity of SVAs and the absence of standardized treatment protocols, further studies are necessary to establish optimal management strategies and long-term surveillance guidelines.

This report describes a single incidental case of SVAs managed conservatively; by nature, a single case cannot establish causality or define optimal management strategies. Our literature review is based solely on published case reports and may be influenced by publication bias, as asymptomatic or unreported cases likely exist. Furthermore, the heterogeneity in diagnostic modalities, follow-up durations, and outcome reporting among the identified cases limits the ability to perform quantitative synthesis or draw firm conclusions. Follow-up for our patient extends to 12 months, but longer-term stability beyond this period remains unknown. Finally, findings derived from isolated case reports may not be generalizable across diverse patient populations or healthcare settings.

## 5. Conclusion

SVAs are rare vascular anomalies, often asymptomatic and discovered incidentally. However, in some cases, they present with compressive symptoms or thromboembolic complications. Diagnosis relies on imaging, particularly CT, MRI, and US. Through a comprehensive review of previously reported cases, our study provides further insight into their clinical presentation and management. Given the absence of standardized treatment guidelines, management should be individualized based on symptom severity and complication risk. Further studies with larger cohorts and longer follow-ups are needed to establish evidence-based protocols and improve patient outcomes.

## Author contributions

**Data curation:** Hamza A. Abdul-Hafez, Mohammed A. Barakat, Mahmoud Alawneh.

**Supervision:** Hamza A. Abdul-Hafez, Alaa Zayed.

**Writing – original draft:** Hamza A. Abdul-Hafez, Alaa Zayed, Abdullah Raed Hawawrah, Mohammed A. Barakat, Alaa Hasan, Mahmoud Alawneh.

**Writing – review & editing:** Hamza A. Abdul-Hafez, Alaa Zayed, Abdullah Raed Hawawrah, Mohammed A. Barakat, Alaa Hasan, Mahmoud Alawneh.

**Investigation:** Mohammed A. Barakat, Mahmoud Alawneh.

## Supplementary Material


