# Supplementary material for: Subclavian vein aneurysm: A case report of a very rare vascular anomaly with a comprehensive literature review
Source: Medicine (Baltimore). 2025 Jul 18;104(29):e43278. doi: 10.1097/MD.0000000000043278 (PMC12282811; doi:10.1097/MD.0000000000043278)
Supplement: Supplementary file 1 [file medi-104-e43278-s001.docx]

Supplementary Table: A comprehensive review of previous reported cases of subclavian vein aneurysm

| # | Author/ year | Age (years) | Gender | Size (cm) | Side | Risk factors and associated diseases | | | | | | | | Presenting symptoms | | | | Physical examination | Histology | | Diagnostic Radiological modality | | | | | | Management | | | Complications | Outcome | Last Follow-up (months) |
| --- | --- | --- | --- | --- | --- | --- | --- | --- | --- | --- | --- | --- | --- | --- | --- | --- | --- | --- | --- | --- | --- | --- | --- | --- | --- | --- | --- | --- | --- | --- | --- | --- |
|  |  |  |  |  |  | HTN | DM | PSH | Trauma | Vasculitis | Genetic diseases | Other vascular anomalies | other | Asymptomatic | Neck pain | Neck swelling | Other |  | Biopsy | Findings | MRI | Findings | CT | Findings | US | Findings | Conservative | Open Surgical | Endovascular |  |  |  |
| 1 | SCHELLHAMMER et al. 2005 | 22 | M | 1.7*0.8 cm | L | No | No | No | No | No | No | No |  | Yes | No | No |  | NA | No | - | No | - | Yes | saccular aneurysm of the left subclavian vein of 17*8 mm diameter located directly at the confluence of the internal and external jugular vein | No | - | Yes | No | No | NA | NA | NA |
| 2 | Elbasty et al. 2018 | 19 | M | 3.1*2.8 cm | L | No | No | No | No | No | No | No |  | No | No | Yes |  | soft supraclavicular mass that was readily compressible | Yes | N/A | No | - | Yes | a 31*28 mm left subclavian vein aneurysm | Yes | a 31*28 mm left subclavian vein aneurysm | No | Yes | No | NA | NA | NA |
| 3 | Choi et al. 2015 | 54 | F | N/A | L | No | No | No | No | No | No | diffuse infiltrative laryngoesophageal and peritoneal venous malformations | Thyroid cancer | Yes | No | No |  | NA | Yes | irregularly dilated thin-walled vascular channels with some fibroadipose tissues | No | - | Yes | focal outpouching sac at the inferior aspect of the left subclavian vein. | No | - | Yes | No | No | NA | Stable | NA |
| 4 | Parizh et al. 2016 | 56 | F | 5 *4 cm | R | Yes | No | No | No | No | No | No | Thyroid disease | No | Yes | Yes |  | an 8*8 cm palpable mass was noted between the right clavicle and the deltoid muscle. The mass was firm with limited mobility on palpation. | Yes | N/A | No | - | Yes | saccular aneurysm of the right subclavian measuring 5*4 cm, containing a large intramural thrombus. | No | - | No | Yes | Yes (failed) | Thrombus | NA | NA |
| 5 | Warren et al. 2013 | 12 | F | 6*4 cm | R | No | No | No | No | No | No | benign hamartomatous tumor of the vessel wall |  | No | Yes | Yes |  | soft, nonpulsatile, nontender mass in the right supraclavicular region | Yes | - large lobules of mature adipose tissue separated by bands of bland fibrous tissue.  - benign hamartomatous lesion consisting of adipose tissue, fibrous tissue and small vessels and nerves, arising from the wall of the subclavian vein and resulting in the aneurysmal changes. | Yes | presence of a lipomatous mass extending circumferentially around the central right subclavian vein, likely arising from the wall vessel. | Yes | long segment fusiform dilation of the right subclavian vein extending laterally from near the junction of the subclavius muscle and first rib to the mid-portion of the vein | Yes | fusiform aneurysm with an associated fatty mass and showed a normal venous waveform | No | Yes | No | NA | NA | NA |
| 6 | Afifi et al. 2012 | 63 | F | 15 *10 cm | L | No | No | haematoma evacuation and ligation of the left internal jugular vein | Yes (10 years ago) | No | No | No |  | No | No | Yes |  | left-sided, soft, pulsating swelling extending from the angle of the mandible to the clavicle (15 × 10 cm) which increased in size significantly on lying flat. | No | - | No | - | Yes | aneurysmally dilated venous channel related to the left subclavian vein. | Yes | hugely dilated venous channel with sluggish whirling flow. | No | No | Yes | No | Recovery | NA |
| 7 | McCready et al. 2007 | 66 | F | 5.1*4.4 cm | L | No | Yes | No | No | No | No | No | CHF, OSA, P.HTN, thyroid surgery | No | Yes | Yes |  | large soft supraclavicular mass that was readily compressible. The mass increased in size with a Valsalva maneuver | Yes | dilated segment of vein. | No | - | Yes | a 5.1 x 4.4-cm venous aneurysm arising from the left subclavian vein | No | - | No | Yes | No | No | Recovery | 8 |
| 8 | Shaked et al. 1984 | 18 | F | N/A | R | No | No | subtotal resection of superior mediastinum mass | No | No | No | lymphangiolipo hamartoma |  | Yes | No | No |  | NA | No | - | No | - | Yes |  | No | - | Yes | No | No | No | NA | NA |
| 9 | Bartline et al. 2015 | 58 | F | 7.1 cm (six years ago, it was 3.9 cm) | R | No | No | No | No | No | NF1 | right innominate and internal jugular veins aneurysm | A.fib, P.HTN, OSA | No | Yes | Yes | 6 years ago, it was asymptomatic. SOB, dysphagia, and hoarseness of voice. | NA | Yes | diffuse neurofibroma with an infiltrative pattern, which stained strongly positive for S100 and CD34 | No | - | Yes | a 7.1-cm venous aneurysm involving the right innominate, jugular, and subclavian veins. | Yes | confirmed the extent of venous involvement and also showed the presence of partially occlusive, age-indeterminate thrombus in the right subclavian vein. | No | Yes | No | NA | Recovery | 2 |
| 10 | San Noberto et al. 2010 | 73 | F | 4.2*3.7 cm | R | Yes | No | No | No | No | No | No |  | No | No | Yes | SOB | right supraclavicular compressible mass that increased in size with a Valsalva maneuver. | No | - | No | - | Yes | a 4.2-cm 3.7-cm venous aneurysm arising from the right subclavian vein with larger intraluminail thrombus | Yes | aneurysm appearing to arise from the right subclavian vein close to the jugular vein. | No | No | Yes(transcatheter coil embolization) | Thrombosis, PE | Recovery | 18 |
| 11 | Roos et al. 2011 | 72 | F | 4.8 × 3.3 × 2.9 cm | L | No | No | No | No | No | No | No |  | No | No | Yes |  | soft, elastic, non-tender mass was palpated in the left supraclavicular fossa. | Yes | brous thickening, proliferated fibrocytes, and capillary ingrowth within the adventitial layers | No | - | Yes | cystic lesion measuring 4.8 × 3.3 × 2.9 cm, located at the left jugulobrachial venous angle. | Yes | well-defined, oval-shaped lesion with an inhomogeneous, hypoechoic to anechoic internal structure | No | Yes | No | No | Recovery | 12 |
| 12 | Mira et al. 2002 | 42 | M | 2 * 2 cm | L | No | No | No | Yes | No | No | No | Left Clavicular fracture | Yes | No | No | during workup of suspected upper lobe lung nodule | Unremarkable | No | - | No | - | Yes | Subclavian and azygous vein aneurysm in setting of lymphangio-lipo hamartoma in superior mediastinum | No | - | Yes | No | No | No | Stable | NA |
| 13 | Ghumman et al. 2024 | 94 | F | 2.6 × 1.3 cm | R | Yes | Yes | No | No | No | No | No | A.Fib, CHF, PAD | Yes | No | No | Presented with right CVA | Unremarkable | No | - | No | - | Yes | focal outpouching of the right subclavian vein measuring 2.6 × 1.3 cm | No | - | Yes | No | No | No | NA | NA |
| 14 | Our case | 37 | M | 2.2 × 2.0 × 1.7 cm | R | No | No | No | No | No | No | No |  | Yes | No | No |  | Unremarkable | No | - | Yes | abnormal rounded signal at the proximal right subclavian vein | Yes | fusiform aneurysmal dilation of the proximal right subclavian vein, measuring 2.2 × 2.0 × 1.7 cm. | No | - | Yes | No | No | No | Stable | 12 |
| M: Male; F: Female; L: Left; R: Right; SVA: Subclavian Vein Aneurysm; CT: Computed Tomography; MRA: Magnetic Resonance Angiography; MRI: Magnetic Resonance Imaging; A. Fib: Atrial Fibrillation; CVA: Cerebrovascular Accident; CHF: Congestive Heart Failure; DM: Diabetes Mellitus; HTN: Hypertension; NA: Not Available/Not Applicable; NF1: Neurofibromatosis Type 1; OSA: Obstructive Sleep Apnea; PAD: Peripheral Artery Disease; P. HTN: Pulmonary Hypertension; PE: Pulmonary Embolism; US: Ultrasound | | | | | | | | | | | | | | | | | | | | | | | | | | | | | | | | |
